# The Relationship between Smoking and Susceptibility to HIV Infection: A Two-Sample Mendelian Randomization Analysis

**DOI:** 10.3390/biomedicines12092060

**Published:** 2024-09-10

**Authors:** Min-Rui Yu, Wei Hu, Song Yan, Meng-Meng Qu, Yan-Mei Jiao, Fu-Sheng Wang

**Affiliations:** 1 Medical School of Chinese PLA, Beijing 100853, China; ymr121331@163.com; 2 Senior Department of Infectious Diseases, The Fifth Medical Center of Chinese PLA General Hospital, National Clinical Research Center for Infectious Diseases, Beijing 100039, China; huwei301yy@163.com (W.H.); qumm302@163.com (M.-M.Q.); 3 Department of Emergency, The Fifth Medical Center of Chinese PLA General Hospital, Beijing 100039, China; 4 Department of Gynecology and Obstetrics, Tangdu Hospital, Air Force Medical University, Xi’an 710038, China; yansong@fmmu.edu.cn

**Keywords:** HIV infection, smoking, two-sample mendelian randomization

## Abstract

Smoking is prevalent among people living with the human immunodeficiency virus (HIV), and it increases morbidity and mortality in this population. However, due to ethical constraints, there is limited information on the effects of smoking on susceptibility to HIV infection. To investigate whether smoking is associated with an increased susceptibility to HIV infection, we conducted a two-sample Mendelian randomization (MR) study using summary statistics from genome-wide association studies of individuals of European ancestry who have ever smoked (n = 99,996) and have HIV (n = 412,130). The random-effects inverse-variance weighted estimation method was used as the study’s primary approach, with the MR-Egger regression and the weighted-median method as complementary approaches. Using 100 single-nucleotide polymorphisms of genome-wide significance as instrumental variables for smoking, we observed a significant association between smoking and HIV infection (odds ratio 5.790, 95% confidence interval [1.785, 18.787], and *p* = 0.003). Comparable results were obtained using the weighted-median method. Our findings implied that smoking is probably associated with increased susceptibility to HIV infection. Given the exploratory nature of this study, further research is needed to confirm this relationship.

## 1. Introduction

The human immunodeficiency virus (HIV) primarily targets CD4+ T cells, leading to acquired immune deficiency syndrome (AIDS) and resulting in an excessive inflammatory response. According to the Joint United Nations Programme on HIV/AIDS, approximately 39 million people worldwide were living with HIV in 2022, with 1.3 million new infections that year (https://www.unaids.org). HIV infection remains one of the most significant public health concerns globally as well as in China [1]. To prevent new infections and control the HIV epidemic, it is crucial to explore the susceptibility factors associated with HIV infection.

Tobacco use is widely recognized as a global health concern; it elevates the risk of cardiovascular diseases, malignancies, infections, and mortality [2]. However, whether smoking increases susceptibility to HIV infection remains unclear. Approximately 42.4% of people living with HIV (PLWH) are smokers, and smoking rates among PLWH are 2–3 times higher than those in the general adult population [3,4]. In PLWH, smoking significantly increases susceptibility to multiple infectious diseases, such as papillomavirus infection and tuberculosis [5,6]. Notably, all-cause and non-AIDS-related mortality rates are higher among PLWH who smoke compared to nonsmokers. In the era of free antiretroviral therapy (ART), the life years lost to smoking in PLWH surpass those lost to HIV [7]. Thus, PLWH are more prone to smoking, and smoking significantly increases the incidence of multiple disorders [5,8], including higher morbidity and mortality [7]. Conversely, smoking cessation enhances the quality of life in PLWH [9]. An initial multiancestry meta-analysis has indicated a correlation between tobacco use disorder and HIV infection [10]. However, there is limited information on the effects of smoking on susceptibility to HIV infection, and whether smoking is associated with an increased susceptibility to HIV infection remains unclear.

In some instances, observed behavioral effects have contradicted clinical observations and prevailing assumptions, potentially due to unmeasured confounders that skewed the perceived causal relationship. Mendelian randomization (MR) analysis uses genetic variants as instrumental variables (IVs) to explore the causal relationships between exposures and clinical outcomes. This approach is less susceptible to confounding factors or reverse causation compared to traditional observational studies [11]. Previous MR studies have found that smoking increases susceptibility to infectious diseases, such as COVID-19 [12,13]. To investigate whether smoking is associated with an increased susceptibility to HIV infection, we utilized data from a large, publicly available genome-wide association study (GWAS) and conducted a two-sample MR study.

## 2. Materials and Methods

### 2.1. GWAS Summary Data Source

The GWAS summary data on smoking exposure were retrieved from the Integrative Epidemiology Unit (IEU) OpenGWAS project (https://gwas.mrcieu.ac.uk/) (accessed on 30 March 2024); they are accessible under the dataset number ieu-b-4858, which represents smoking. This dataset includes a total of 99,996 samples and 7,933,821 single nucleotide polymorphisms (SNPs), all from European populations. We obtained published GWAS meta-analysis data on HIV infection from FinnGen Release R10 (https://www.finngen.fi/en) (accessed on 30 March 2024). FinnGen is a significant public research initiative that has gathered and analyzed genetic and health data from 500,000 European donors in Finnish biobanks. The specific dataset we accessed represents PLWH and consists of 412,130 samples. All the utilized data are summarized data.

### 2.2. IVs Selection

Based on the Strengthening the Reporting of Observational Studies in Epidemiology Using Mendelian Randomization (STROBE-MR) guidelines [14], we performed an MR analysis to investigate the association between smoking and susceptibility to HIV infection. This MR design assumed (1) that the genetic variants were associated with smoking, (2) that there was no association between the genetic variants and any confounding factors of HIV infection, and (3) that the genetic variants affected HIV infection solely through smoking. To establish a robust inference, we applied the following screening criteria for IV selection: First, the SNPs were associated with the targeted exposure at a genome-wide significance threshold of *p* < 10^−5^. Second, SNPs in an linkage disequilibrium (LD) clump window of less than 10,000 and an r^2^ greater than 0.001 were excluded. Third, F-statistics for each SNP were calculated using the formula F = β^2^/Se^2^. SNPs with F-statistics below 10 were considered weak IVs and were thus excluded from subsequent analyses. Fourth, to further exclude the impact of confounding factors, such as natural HIV control, HIV disease progression, and viral load set point, we searched the LDstrait database (https://ldlink.nih.gov/?tab=ldtrait) (accessed on 2 April 2024) and related GWASs [15,16].

### 2.3. MR Analysis

All the original studies obtained ethical approval and secured informed consent from participants. The data utilized in this analysis were anonymized and are publicly available; this removes the need for additional ethical approval and informed consent for the present study.

After screening for SNPs present in both the exposure and outcome data, a harmonization operation was performed to remove any palindromic structures. Then, an MR analysis was conducted. The effects of each IV were calculated using the Wald ratio, which corresponds to the log odds ratio for the outcome per unit change of the exposure. This method is the simplest for estimating the causal effect of the exposure on the outcome and is derived by dividing the coefficient of the genetic variant in the regression of the outcome by the coefficient of the genetic variant in the regression of the exposure [17]. Three methods were used to summarize the total effects of the included IVs and infer the association between smoking and HIV infection: the random-effects IVW method, the MR-Egger regression, and the weighted-median estimator. The IVW method was chosen as the primary analytical approach according to the guidelines for performing MR investigations [18,19]. To assess the robustness of the findings, we performed a sensitivity analysis. The MR-Egger regression, which employs summarized genetic data, was used in this study to establish inferences and detect pleiotropy among IVs [20]. The weighted-median estimator provides consistent association estimates even if up to half of the data come from invalid genetic variants [21].

### 2.4. Sensitive Analysis

To assess the robustness of our MR results and to determine whether the results were biased or if any IV strongly influenced the outcome, we conducted several sensitivity analyses. Initially, owing to potential heterogeneity caused by differences in sample acquisition and sequencing batches among European populations, we performed heterogeneity tests on the results of the IVW and MR-Egger methods. A *p*-value greater than 0.05 indicated no significant heterogeneity. In addition, funnel plots were utilized to assess potential asymmetry in SNP distribution relative to the IVW line, focusing on whether the data points on the left and right sides were approximately symmetrical. If outliers were identified, they could be removed, allowing the MR analysis to be repeated to ensure the robustness of the results. Furthermore, to address the possibility of spurious noncausal associations due to pleiotropy, we employed MR-Egger regression to test for significant horizontal pleiotropy, considering *p* < 0.05 as indicative of significant pleiotropy. The MR-Pleiotropy RESidual Sum and Outlier (MR-PRESSO) test is an advanced statistical method designed to detect and correct potential pleiotropic effects in MR analyses. It enhances the accuracy and reliability of MR analysis results by identifying and handling SNPs that may influence outcomes through pathways unrelated to the studied exposure factor. Significant pleiotropy implies that the IVs are related not only to the exposure factors but also to other confounding factors [22]. Finally, we performed a leave-one-out sensitivity analysis. This involved sequentially excluding each SNP, recalculating the meta-effect of the remaining SNPs, and observing if the results varied significantly after the exclusion of any particular SNP. The consistency of the error lines—whether all to the right or left of 0—suggested that the results were robust.

### 2.5. Molecular Pathways Connecting Smoking and HIV Infection

To explore the molecular functions regulating the relationship between smoking and HIV infection, the NCBI-Batch Entrez tool (https://www.ncbi.nlm.nih.gov/sites/batchentrez) (accessed on 3 May 2024) and the STRING database (https://string-db.org) (accessed on 3 May 2024) were utilized to construct a protein–protein interaction (PPI) network and perform functional enrichment analysis.

### 2.6. Statistical Analysis

The MR analyses were performed using the TwoSampleMR package (version 0.5.8), MRInstruments (version 0.3.0), and dplyr (version 1.1.4) in R (version 4.3.1). Furthermore, ggplot (version 3.5.1) was used to visualize the STRING enrichment results. Statistical significance for the MR-effect estimates was set at *p* < 0.05.

## 3. Results

### 3.1. Characteristics of the Genetic Instruments

**Figure 1:**
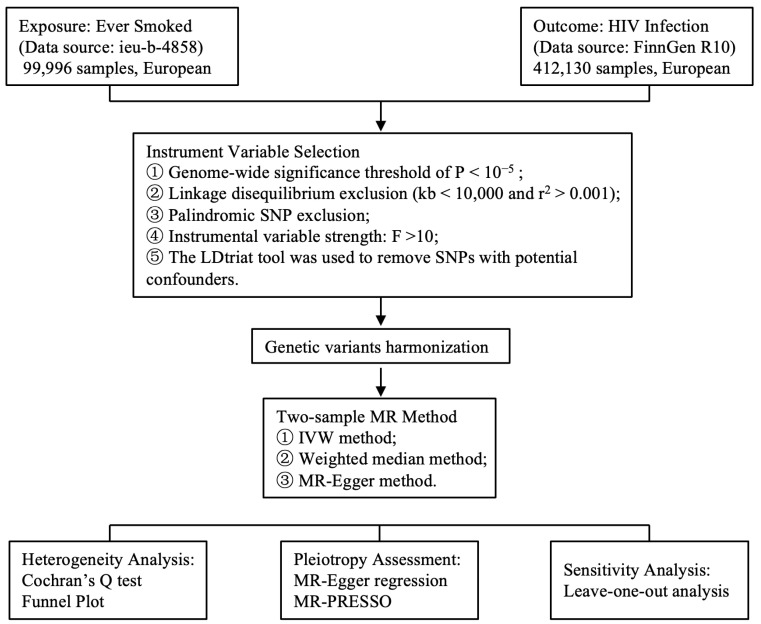
Flowchart of the MR.

To explore whether smoking is associated with an increased risk of HIV infection, we conducted two-sample MR tests. As illustrated in Figure 1, the process began by downloading smoking- and HIV infection-related datasets from public databases. Sets of IVs for smoking were established using predefined screening criteria. Weak (F statistic < 10) and insignificant (*p* ≥ 10^−5^) IVs, as well as SNPs in LD (r^2^ > 0.001 within LD distance < 10,000), were excluded from current MR study. Next, the SNPs were harmonized to ensure alignment at the same gene loci for both the exposure and outcome data. As a result, we identified 100 independent instruments associated with smoking for MR analysis. None of the IVs was further removed since the 100 IVs were not associated with HIV infection, disease progression, viral load set point, or other potential confounders. MR analysis was then performed using three methods to infer the genetic liability of smoking on susceptibility to HIV infection. Finally, the robustness of the inference was assessed.

### 3.2. Effect of Smoking on HIV Infection

**Figure 2:**
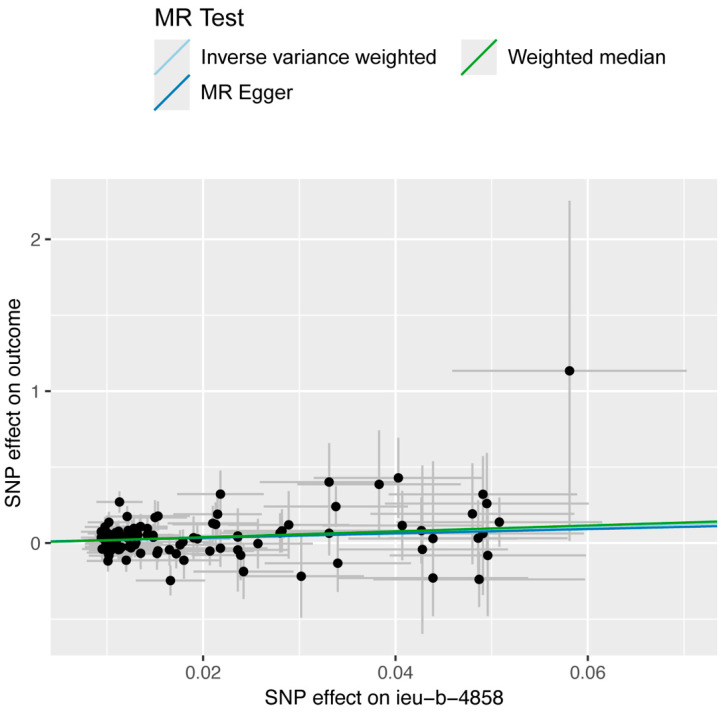
Scatter plots of MR of smoking on susceptibility to HIV infection. The trait on the *x*-axis denotes the exposure, while the trait on the *y*-axis denotes the outcome. Each point represents a single IV. The dots and the short lines through the dots denote the effect sizes and the 95% CIs of smoking on HIV infection. The slopes of the lines illustrate the effect ratios of exposure to outcome. The results, estimated by different methods, are distinguished by the colors of the lines.

**Figure 3:**
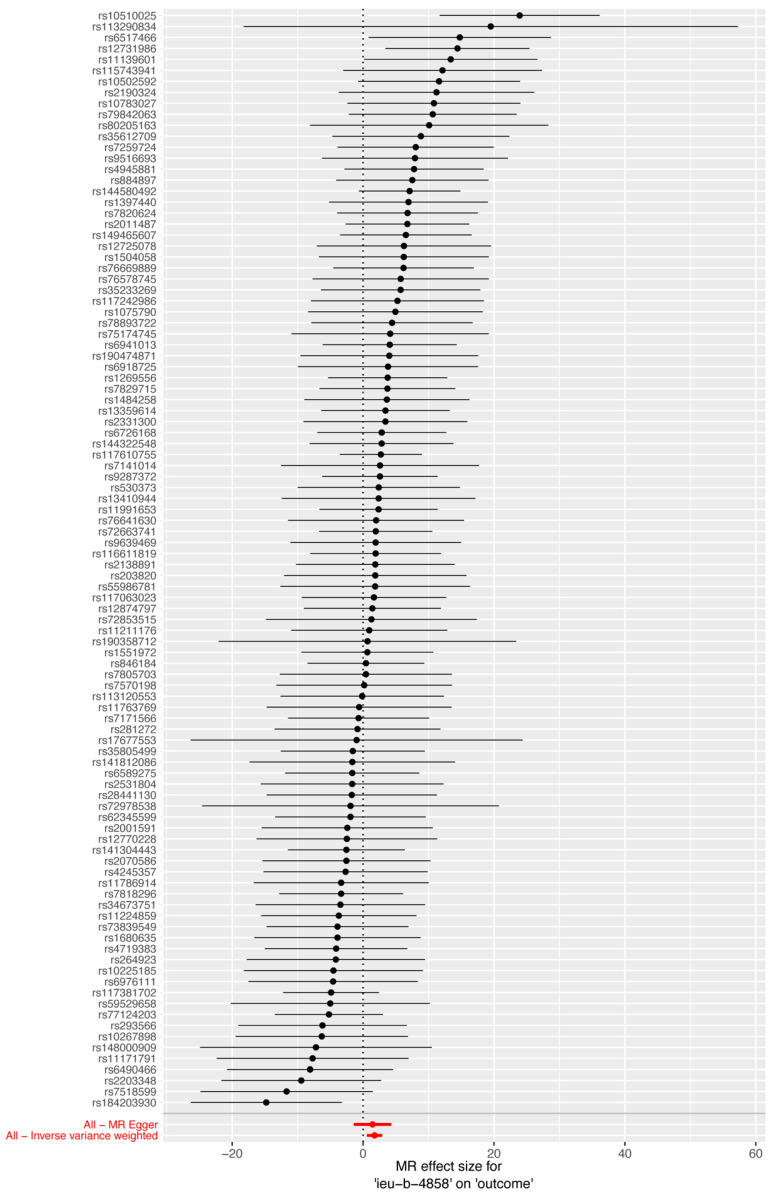
Forest plot of MR of smoking on susceptibility to HIV infection. Each horizontal line represents an individual SNP’s estimate using the Wald ratio method. The red line at the bottom summarizes the overall results: smoking is associated with increased susceptibility to HIV infection.

**Figure 4:**
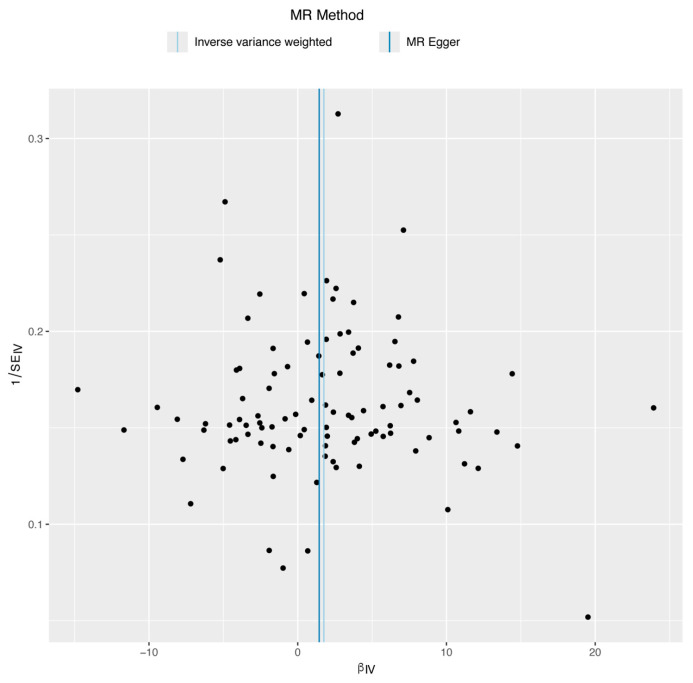
Funnel plot of MR of smoking on susceptibility to HIV infection.

**Figure 5:**
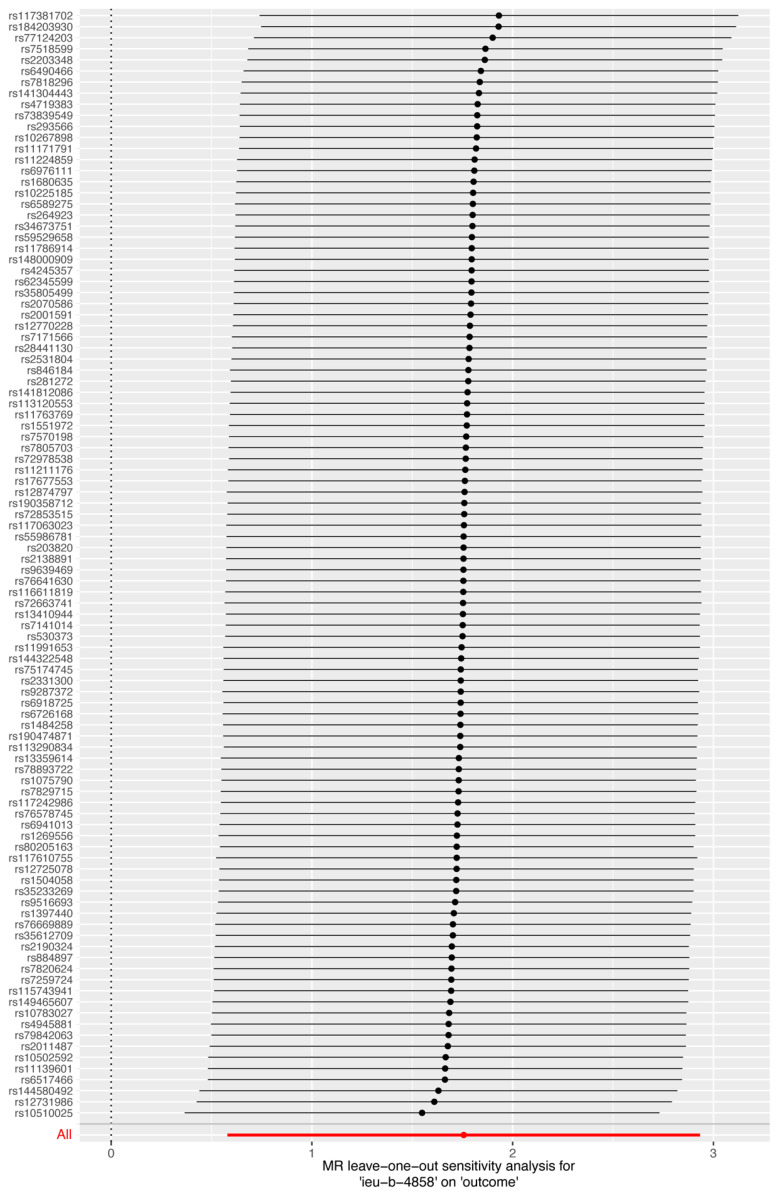
Leave-one-out sensitivity analysis for MR of smoking on susceptibility to HIV infection. After removing each single SNP, the overall error bars remained consistent, and all the bars were to the right of zero, thereby supporting the robustness of our findings.

The MR analysis is presented in Table 1 and Figure 2, Figure 3, Figure 4 and Figure 5. In Figure 2, we present the results of the MR analysis of the relationship between the exposure factor (smoking) and the outcome factor (HIV infection). The slopes of the colored lines, which represent different statistical methods, illustrate the effect ratios of exposure to outcome. The upward slant of these lines suggests that increased smoking exposure raises the risk of HIV infection. Table 1 shows that smoking was significantly associated with increased susceptibility to HIV infection, except when using the MR-Egger method. For the IVW method, the statistics are as follows: β = 1.756, odds ratio (OR) 5.790, 95% CI [1.785, 18.787], and *p* = 0.003. For the weighted-median method, the statistics are as follows: β = 1.928, OR 6.876, 95% CI [1.166, 40.556], and *p* = 0.033. 

Figure 3 shows a forest plot of the SNPs, with each horizontal line representing an individual SNP’s estimate using the Wald ratio method. The red line at the bottom summarizes the overall results: smoking is associated with increased susceptibility to HIV infection.

### 3.3. Sensitivity Analysis

After obtaining the results of the MR analysis, we evaluated their robustness through several statistical tests. The IVW result was assessed for heterogeneity using the Cochran Q test (*p* = 0.646), which indicated no significant heterogeneity (detailed results can be found in Supplement S1). A funnel plot (Figure 4) displays that the points on both sides of the IVW and MR-Egger regression lines are approximately symmetrical, initially suggesting balanced pleiotropy. Subsequently, we conducted MR-Egger regression and found no significant pleiotropy (*p* = 0.815; for detailed results, see Supplement S2). Consistent estimates were obtained from the MR-PRESSO test (*p* = 0.631; detailed results can be found in Supplement S3). Finally, we conducted a leave-one-out analysis by sequentially eliminating each SNP to observe changes in the meta-effect of the remaining SNPs. Significant changes after the removal of a specific SNP would indicate a substantial influence on the results and the presence of outliers. As demonstrated in Figure 5, after removing each SNP, the overall error bars remained consistent, and all the bars were to the right of zero, thereby supporting the robustness of our findings.

### 3.4. Bioinformatical Analysis

**Figure 6:**
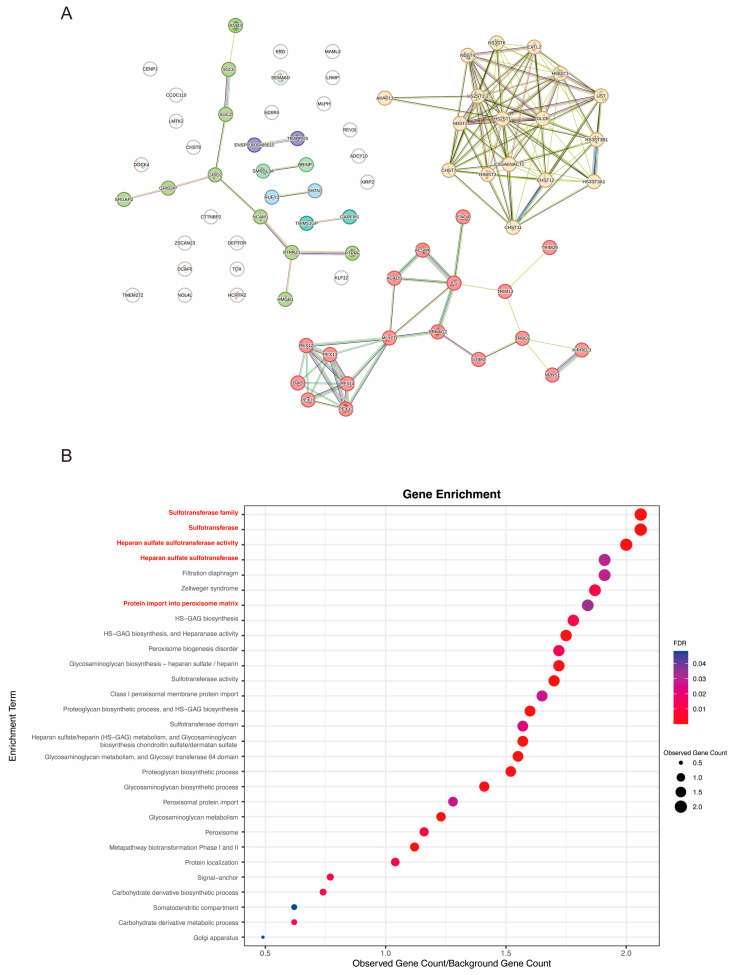
Bioinformatical analysis of the identified candidates in the MR of smoking on susceptibility to HIV infection. (**A**) The PPI of identified candidates in the MR of smoking on susceptibility to HIV infection. The line sizes are proportional to the combined scores of the interactions. (**B**) Dot plot of the functional enrichment results. The sizes of the dots are proportional to the number of genes analyzed; the larger the dots, the more genes enriched in the enrichment term. The color represents the FDR; the stronger the red, the smaller the value of the FDR. PPI, protein–protein interaction; FDR, false discovery rate.

We identified 100 SNPs, along with their corresponding traits and potential genes, using the LDstrait tool. Utilizing the STRING database, we constructed a PPInetwork with these genes, as depicted in Figure 6A. The network comprised 76 nodes and 144 edges, with an average node degree of 3.79. These proteins were organized into seven distinct clusters via K-means clustering, indicating potential interactions between these genes. Subsequently, these genes were used for enrichment analysis in STRING. As shown in Figure 6B, the top three enriched terms were sulfotransferase family, sulfotransferase activity, and heparan sulfate (HS) sulfotransferase activity. These characteristics are associated with HIV-1 attachment and intercellular dissemination [23].

## 4. Discussion

In this study, we found that smoking is associated with increased susceptibility to HIV infection. Furthermore, 100 SNPs were identified as mediators in the association between smoking and HIV infection, with enrichment in the sulfotransferase family, sulfotransferase activity, and HS sulfotransferase activity.

In the post-ART era, lifestyle-related factors may represent a greater risk for PLWH mortality than the direct impacts of the HIV virus [24]. The smoking rate of PLWH is higher than that of the HIV-negative population, possibly due to responses to HIV infection-related symptoms such as neuropathic pain, anxiety, and depression [5]. PLWH are in a state of immunosuppression and are more likely to suffer from tobacco-related diseases. A case-control study of ART-naive PLWH found that smokers were three times more likely to develop pulmonary tuberculosis than nonsmokers [25]. Bacterial pneumonia is highly prevalent among PLWH, with those receiving ART and currently smoking being three times more likely to develop bacterial pneumonia compared to nonsmokers [26]. Another study found that the risk of smoking-related cancers among PLWH was twice as high in former smokers compared to nonsmokers [8]. Compared to nonsmokers, smokers with HIV infection showed significantly higher activation of CD4+ and CD8+ T cells, which promoted systemic inflammation [27]. In addition to tobacco-related diseases and inflammation, smoking also contributes to HIV replication and viral rebound. Smoking was found to be associated with a higher HIV viral load, with PLWH who had smoked in the past 30 days being 1.5 to 2 times more likely to have an HIV viral load above 100,000 cp/mL while on ART compared to nonsmokers [28]. These findings indicate that smoking significantly increases the incidence of multiple disorders in PLWH and is detrimental to viral control. However, the association between smoking and susceptibility to HIV infection remains unclear.

Smoking has been shown to increase the prevalence of multiple infectious diseases [29]. For instance, smokers have approximately twice the rate of severe infectious respiratory diseases compared to nonsmokers [30]. A recent MR study supported this view, indicating that smoking increases susceptibility to respiratory infections [13]. Another study reported that susceptibility to influenza infection is approximately 2.5 times higher in smokers than in nonsmokers [31]. Additionally, mice exposed to cigarette smoke (CS) extract had a higher incidence of influenza B virus infections and increased mortality post-infection [32]. The potential mechanisms might include compromised primary antiviral immunity, such as decreased influenza B virus-specific IgG levels [32]. An in vitro study found that CS exposure promotes rhinovirus infection in human bronchial epithelium by altering the expression of interferons and inflammation-related genes [33]. Regarding the association between smoking and COVID-19, several MR studies have demonstrated strong links between smoking and COVID-19 infection [12,13]. These findings are consistent with a community-based cohort study [34]. However, due to ethical constraints and the unavailability of suitable animal models, there is limited information on the effects of smoking on susceptibility to HIV infection. So far, only an initial multi-ancestry meta-analysis has indicated a correlation between tobacco use disorder and HIV infection [10]. In our study, we provide genetic evidence showing that smoking is associated with increased susceptibility to HIV infection (OR 5.790, 95% CI [1.785, 18.787], *p* = 0.003).

Existing studies have shown that the frequency of HLA-B*57 alleles is significantly increased in HIV-1 elite controllers and that genetic variation in the major histocompatibility complex (MHC) region has the greatest genetic impact on viral load control and HIV progression [35,36,37]. However, the question of whether the HLA family affects HIV susceptibility is still unclear and requires further research. In this study, the LDstrait tool revealed that the SNP rs1269556 was associated with MHC class I polypeptide-related sequence A levels and metabolic factors such as body mass index and urate levels [38]. This suggests a complex interplay between smoking behavior, immune-inflammatory responses, and metabolic factors. For instance, variations in the HLA family modulate immune function [39], thus influencing not only HIV replication and infection progression but also HIV entry, establishment, and susceptibility. However, further research is needed to confirm whether variations within the HLA family influence HIV susceptibility and to elucidate the potential mechanisms involved.

Smoking negatively impacts both innate and adaptive immunity, modifying immune responses by intensifying harmful immunological reactions and/or suppressing defense immunity [40]. In a mouse model of emphysema, exposure to CS restricted the antiviral innate immune response and impaired the activation of adaptive CD8+ T cells; these results support the view that smoking is a major driver of compromised antiviral capabilities [41]. Jie Chen et al. [42] found that CS interferes with the generation and presentation of MHC class I antigens, resulting in impaired activation of CD8+ T cells during viral infections. Ying Shao and colleagues [43] discovered that long-term exposure to a combination of CS and morphine significantly reduced the number of CD4+ Treg cells. In addition to affecting T cells, CS exposure can significantly influence the proportions of B-cell subsets in peripheral blood [44]. Smoking-related immune abnormalities include decreased circulating IgG concentration and decreased production of specific IgG antibodies against respiratory and nonrespiratory pathogens, which raises the risk of viral infection [40,45]. The SNPs used in the present study are associated with antiviral (rs6589275) [46], antibacterial (rs7141014) [47], and antifungal (rs7141014) [48] immunity.

Smoking induces an imbalance in innate immunity. CS activates caspase-1 in human macrophages, which leads to macrophage dysfunction and increases the risk of infection [49]. Another study found that exposure to CS could lead to the activation of caspase-1, -4, and -8, as well as the cleavage of gasdermin D, which resulted in macrophage dysfunction and exacerbated chronic inflammation in the lungs of smokers [50]. The proinflammatory cytokines interleukin (IL)-1α and IL-1β were significantly elevated in the lung-tissue and sputum samples of patients with chronic obstructive pulmonary disease who smoked compared to those of nonsmokers [51]. Among the SNPs used in the present study, rs530373 and rs1269556 are associated with levels of the tumor necrosis factor receptor superfamily, member 9 [52]; rs2011487 is linked to natural killer cell functions [53]; rs7141014 and rs28441130 are associated with lymphocyte count [54]; and rs11211176, rs9287372, rs6918725, and rs34673751 are linked to white blood cell count [55]. These data suggest that altered immune responses may mediate the relationship between smoking and increased susceptibility to HIV infection.

The PPI-enrichment analysis revealed that the associated genes of these SNPs were enriched in the sulfotransferase family, sulfotransferase activity, HS sulfotransferase activity, protein import into the peroxisome matrix, and proteoglycan biosynthetic process. HS chains—large anionic polysaccharides from the glycosaminoglycan family—significantly influence adhesion and transport across epithelial layers, chemotaxis, tissue invasion, and cellular entry into mucosal cells, which serve as portals for HIV-1 entry into the body. Before the successful establishment of infection, HS has been shown to play a crucial role in HIV adsorption and spread [23,56]. Furthermore, cell surface proteoglycans have been identified as the main HIV-1 receptors on primary human endothelial cells, and proteoglycans have been demonstrated to facilitate HIV-1 attachment and/or entry into CD4-negative cells. Proteoglycans may also be involved in the intercellular dissemination of HIV-1 [57]. Previous studies have reported that smoking can influence mucosal immunity. For example, CS reduces mucosal vaccine responses by decreasing plasmacytoid dendritic-cell activation and, consequently, Th1-dominant immunity [58]. In smokers, the mucosal metabolomic profile and microbiota are significantly altered [59,60]. Thus, smoking might influence HIV entry, invasion, and infection by affecting mucosal barrier function. The potential mechanisms could involve modulation of the sulfotransferase family, sulfotransferase activity, and HS sulfotransferase activity, which need to be confirmed in animal models.

In summary, our study supports that smoking is probably associated with increased susceptibility to HIV infection in the European population. However, this study has certain limitations. Currently, only GWAS data from the European population are available; hence, more comprehensive studies of different ethnic groups are necessary. A greater sample size is required to more precisely assess the genetic impact of smoking on HIV infection. Moreover, the underlying mechanisms warrant further investigation. The IV analysis indicates that this relationship may be mediated through the antiviral immune response and inflammation associated with mucosal barrier function.

## 5. Conclusions

Our two-sample MR study found that smoking may be associated with increased susceptibility to HIV infection. Further enrichment analysis suggested that this association might be attributed to altered mucosal factors and immune responses, warranting further investigation. Programs targeting smoking cessation and prevention might potentially reduce susceptibility to HIV infection and contribute to the goal of ending AIDS by 2030.

## Figures and Tables

**Table 1:** MR results of the genetic liability of smoking on susceptibility to HIV infection.

Methods	Regression Coefficient (SE)	OR (95% CIs)	N of IVs	*p*	Q	*p* for Q
Inverse variance weighted	1.756 (0.600)	5.790 (1.785, 18.787)	100	0.003	93.182	0.620
MR Egger	1.444 (1.462)	4.237 (0.241, 74.448)	100	0.326	93.127	0.646
Weighted median	1.928 (0.905)	6.876 (1.166, 40.556)	100	0.033	NA	NA

SE: standard error, OR: odds ratio, IVs: instrumental variables, CIs: confidence intervals, NA: not applicable.

## Data Availability

All relevant data are within the manuscript and the supplementary materials. The GWAS summary data are available in the Integrative Epidemiology Unit (IEU) OpenGWAS project (https://gwas.mrcieu.ac.uk/) and FinnGen Release R10 (https://www.finngen.fi/en).

## Supplementary Materials

The following supporting information can be downloaded at: https://www.mdpi.com/article/10.3390/biomedicines12092060/s1. Supplement S1: Heterogeneity in the MR results; Supplement S2: Pleiotropy in the MR results; Supplement S3: MR-PRESSO analysis of the MR results.
